# Retraction: Effect of Cyanotoxins on the Hypothalamic–Pituitary–Gonadal Axis in Male Adult Mouse

**DOI:** 10.1371/journal.pone.0303322

**Published:** 2024-05-02

**Authors:** The PLOS ONE Editors

Following publication of this article [1], concerns were raised regarding multiple figures. Specifically:

- The distribution of the data from some treatment groups in Fig 1, 2A, 2B, 2C, and 5B appears similar despite being generated from different experimental protocols.
- Fig 5B and Fig 6B appear similar.
- The reduced testosterone secretion following decreased GnRH and pituitary Lhβ production would be expected to be associated with an increase in Kiss1 expression in the brain, which is contrary to results reported in [1].
- The magnitude and rapidity of the effects of MC-LR treatment on LH and testosterone levels and subsequent spermatogenesis reduction are in line with what would be expected for complete knockout of the respective hormones, as opposed to a reduction only in the levels reported.
- In the methods, the reported sensitivity of the LH and FSH assays is recorded in IU/L; however, the data reported in Fig 2B are recorded in ng/mL.
- For Figs 5 and 6, the methods suggest quantitative real-time PCR was used to calculate mRNA expression. However, the legends for these figures do not clearly specify which PCR method was used to generate the quantitative data provided. Additionally, the bands in Figs 5A and 6A do not appear to correspond to the data presented in the corresponding histograms in Figs 5B and 6B.

In response to the above concerns, the corresponding author stated that Fig 6B is incorrect. The underlying data for Figs 1, 2A, 2B, 2C, and 5B were not provided for editorial review and therefore concerns regarding these figures could not be resolved. Regarding the remaining issues, the responses from the corresponding author were reviewed by a member of the *PLOS ONE* Editorial Board, who advised that they were not sufficient to resolve the above concerns.

In light of the concerns affecting multiple figure panels that question the reliability of these data, the *PLOS ONE* Editors retract this article.

The corresponding author HX did not respond to the final editorial decision. XX and AZ either did not respond directly or could not be reached.
